# Non-Adherence and First Line Antiretroviral Therapy failure among people living with HIV on Antiretroviral Therapy in Bulawayo city, 2016

**DOI:** 10.4314/ahs.v26i2.4

**Published:** 2026-06

**Authors:** Martin Tatenda Dzimbanhete, Gerald Shambira, Mufuta Tshimanga, Notion Tafara Gombe, Zanele Hwalima, Chiedza Sibanda, Cleophas Chimbetete

**Affiliations:** 1 Department of Community Medicine, University of Zimbabwe; 2 Bulawayo City Health Services Department; 3 Newlands Clinic

**Keywords:** Non-adherence, First line Antiretroviral Therapy Failure, Bulawayo City

## Abstract

**Background:**

The prevalence of first-line ART failure in Bulawayo City, Zimbabwe's second-largest city for the year 2016 was 6.6% higher than the national average of 3.3%. We assessed the association between non-adherence and first-line ART failure persons on ART in Bulawayo City.

**Methods:**

A 1:1 case-control study was done to measure the association between adherence and other risk factors for treatment failure among 169 cases and 169 controls. The Morisky Medication adherence 8-item scale was used to assess self-reported adherence, and non-adherence was defined as having a self-reported score of 1 to 8. Epi info 7 was used for univariate, bivariate, and multivariate analysis.

**Results:**

Persons who were non-adherent to ART were 5 times more likely to experience treatment failure than those who adhered association between non-adherence and treatment failure (OR=5.1, 95% CI 2.9-8.9). The study established that 58% of the cases had good knowledge of adherence to ART compared to 85% of the controls. Stopping medication when feeling worse (AOR=3.5, 95% CI 1.8-7.0) was the most significant independent risk factor for treatment failure.

**Conclusions:**

The study found a strong association between non-adherence and treatment failure. Routine monitoring at every clinic visit of clients on ART should be done to monitor the client's adherence to medication.

## Background

WHO defines treatment adherence as ‘the degree to which a person's behaviour of taking medication corresponds with the health care provider's prescription^1^. Poor adherence has been reported in areas where food has been scarce, and where clients have to travel long distances to seek medical care and is one of the major causes of HIV treatment failure^2^.

HIV treatment failure has three definitions, namely: clinical, immunological and virological. According to the Zimbabwean ART Guidelines, clinical failure in adults is defined as a new or recurrent clinical event indicating severe immunodeficiency (WHO clinical stage 3 and 4 conditions) after 6 months of effective treatment^1^. HIV treatment failure is also defined as a CD4 count which falls below the pre-therapy baseline or there is a 50% fall from the treatment peak value (if known) or CD4 count levels are persistently (at least 2 results) below 100cells/mm3^1^. Virological failure is defined as plasma viral load above 1000 copies/ml based on two consecutive viral load measurements after 3 months, with adherence support^1^. The United Nations declared to end the HIV pandemic by the year 2030 by ensuring that 90 % of all people living with HIV are aware of their status, 90 % know their status being on antiretroviral therapy and 90% of people living with HIV on antiretroviral therapy achieving viral suppression^3^. In a study done in Gabon, the rate of virologic failure on first-line ARV was 41.3% (36.4-46.4)^4^. As a proxy for first-line ART failure, the National HIV Drug Resistance Monitoring survey at 12 sentnel sites in Zimbabwe, from the period of 2009 to 2011 found that the proportion of clients with HIVDR at 12 months, after being initiated on ART was 3.3%^5^.

A case-control study done in Kenya established that baseline CD4 count < 50/ml (H.R = 7.07, (95% CI = 4.92 - 10.15) was significantly associated with ART failure^6^. According to Chimbetete et.al (2011), the most common side effect of antiretroviral therapy among HIV-positive clients was peripheral neuropathy^7^. Dizziness, skin rash, and vomiting were more frequently reported signs and symptoms among the non-adhering clients^7^.

Bulawayo City Council Health Facilities had 10,177 clients in care receiving ART by the end of the first quarter of 2016. The number of clients who failed first-line ART was 716(6.6%), which was two times higher than the national average of 3.3%. A review of the clients' OI/ART registers from the 16 clinics showed that 233(2.3%) of the 716 clients that failed first-line ART in Bulawayo City were from Emakhandeni District, 225 (2.2%) were from Northern Suburbs District and 258(2.5%) were from Nkulumane District. According to the Early Warning Indicator Survey Report of 2013, the adult ART sites in Bulawayo City failed to meet the desired target of on-time pill pick-up of ≥90%^8^. Failure to optimize on first-line regimen results in more clients switching to second-line ART. Second-line ART has more metabolic complications as compared to first-line ART and can lead to the development of chronic conditions such as hypertension^7^. The cost of second-line ART in Zimbabwe ranges from US$30- US$40 per person per month. Contrary, the cost of first-line ART ranges from US$10-US$12 per person per month in 2016. Therefore an investigation was done to find out possible factors of non-adherence and first-line treatment failure in Bulawayo City.

## Methods

### Study design

A 1:1 unmatched case-control study was done to measure the association between adherence and other risk factors for treatment failure among HIV-positive patients on ART in Bulawayo City. A case was defined as a client aged 18 years old and above who was on first-line ART for at least six months in Bulawayo City and switched to a second-line ART regimen owing to treatment failure from January 2015 to June 2016. A control was defined as a client aged 18 years and above from Bulawayo City who is on first-line ART, has been on first-line ART for at least 6 months and has not failed first-line ART from January 2015 to June 2016.

### Sample size and sampling procedure

The Fleiss formula which is embedded in Stat Cal of the Epi Info 7 software, was used to calculate the sample size. The calculation of the sample size was based on a study by Shigdel et.al (2014) on “Factors associated with adherence to antiretroviral therapy in HIV-infected clients in Kathmandu District, in Nepal, India”^9^. The calculated sample size for primary respondents was 169 cases and 169 controls.

The study was done at Bulawayo City Health facilities in Northern Suburbs, Nkulumane, and Emakhandeni Districts. The total numbers of health facilities in Bulawayo City were stratified according to three districts in Bulawayo City. Fourteen health facilities from the three districts were proportionately sampled. For the selection of cases from Bulawayo City, from the clinics, they were proportionally sampled according to the number of clients that failed first-line ART and the OI/ART Clinic attendance register was used as the sampling frame. Cases were randomly selected using the lottery method until a sample size of 169 cases was attained. The same procedure was repeated for the controls until a sample size of 169 controls was achieved. Five key informants were purposively selected and they included expert clinicians, the pharmacist, primary counsellors and expert patients.

### Data collection

A structured pretested interviewer-administered questionnaire was used to collect data from the study participants. The questionnaires were designed in English language and then translated to Ndebele language which is the local language for the study population. Medication adherence was measured using the Morisky Medication Adherence 8th item Scale (MMAS-8) which is a validated tool from Dr. Morisky^10^. Non-adherence was defined as having a self-reported adherence score of 1-8. An interviewer-administered questionnaire was used to collect data from the key informants. A desktop review of 338 patient records was done to collect data on adherence to ART.

### Data analysis

Data were analysed using Epi Info 7 to generate frequencies, means proportions and odds ratios. Using a 95% confidence interval, we performed a bivariate analysis to assess associations between different exposures and first-line ART failure. A p-value of < 0.05 was deemed statistically significant in this analysis. Multivariate analysis by way of a stepwise forward logistic regression model was performed on significant variables that were found in bivariate analysis. Data from the key informants was analysed qualitatively with the use of themes.

### Definition of variables

#### Dependent variable

The dependent variable for the study was treatment failure on first-line ART. It was defined as CD4 count falls below pre-therapy baseline or CD4 count levels are persistently (at least 2 results) below 100cells/mm3 or new or recurrent WHO stage 4 condition in a client who has been on anti-retroviral therapy (ART) for at least 6 months.

#### Independent variables

Independent variables included socio-demographic characteristics such as age (completed years), Gender (male or female), marital status, highest level of education and place of residence (low density and high density). Patient-related factors included the number of pills per day, alcohol intake (yes or no) and having insufficient food (yes or no). Variables for measuring adherence included, sometimes forgetting to take medicine (yes or no) and having difficulties in remembering to take medicine (yes or no). Lastly, variables for measuring knowledge of ART included, that for medicine to work one must not miss a dose (yes or no) and ART to be taken for life (yes or no).

## Results

### Socio-demographic Characteristics of study participants

A total of 169 cases and 169 controls participated in the study. Cases and Controls were statistically comparable in terms of socio-demographic characteristics except on place of residence where more cases were found to be from the high-density areas of Bulawayo City.

### Association between adherence and treatment failure among people on ART

Those who were non-adherent were 5 times more likely to fail first-line ART compared to those who were adherent to ART medication (OR=5.1, 95% CI, 2.9-8.9). The association between non-adherence and treatment failure was statistically significant.

### Patient-related factors and first-line treatment failure among people on ART

Significant patient-related risk factors associated with first-line treatment failure among people on ART were; stopping taking medication when feeling worse (OR=3.5, 95% CI, 1.9-6.6), forgetting to take medication when travelling (OR=2.3, 95% CI 1.2-4.5) and baseline CD4 count of less than 50 cells/mm3 (OR=2.7, CI 1.5-4.9). Other patient-related factors that were associated with first-line treatment failure among people on ART were; drinking alcohol while on ART (OR=2.0, 95% CI 1.2-3.4), smoking marijuana (OR=1.7, 95% CI 0.8-3.6), having insufficient food (OR=2.1, 95% CI 1.3-3.2) and taking other tablets besides ART (OR=1.7, 95% CI 1.0-2.7). However, counselling before the start of treatment was protective from treatment failure (OR=0.5, 95% CI 0.09-2.7).

One of the key informants who was an expert client said that some of the patient-related factors that made them develop treatment failure were engaging in unprotected transactional sex with multiple partners because of the economic situation in the country. Furthermore, the other reason that was stated by the key informant was that some of the clients stopped taking ART when they migrated to neighbouring countries such as South Africa and Botswana to seek employment opportunities. The following are the actual words that were said by the key informant:

*“The socio-economic factors for non-adherence and treatment failure are poverty and unemployment as a result of the macro-economic situation in Zimbabwe. This results in some of us engaging in unprotected transactional sex with multiple sexual partners. We want to pay for our rent and send our children to school. There is unemployment in Bulawayo and this has led some clients to migrate to neighbouring countries like South Africa and Botswana. Some of us stop taking medication when we have travelled out of the country. We are failing to get a sufficient supply of food and a balanced diet due to the socio-economic situation in the country. The medication we take makes us feel hungry and dizzy. Some clients complain of side effects such as dizziness, nausea and vomiting and hallucinations”*

The second key informant who was a health worker stated that most men come late for ART initiation and their acceptance and disclosure of their HIV status to their partners was poor. Furthermore, the second key informant underscored that some male patients who are on ART abused alcohol.

The following are the actual words that were said by the second key informant:

*“Most men come late and are initiated on ART when their CD4 is <50 cells/mm3, disclosure and acceptance of HIV status is still poor. Drug fatigue has been noticed in clients aged 20-35 years old. Alcohol abuse has also been seen from male clients”.*

### Client's knowledge of adherence to ART, Bulawayo City 2016

Client's knowledge of ART medication and perceived effectiveness among PLWHIV was assessed, using the 5-point Likert scale where a scale of 0-2 was poor knowledge, 3 was fair knowledge and 4-5 was good knowledge. Eighty-five per cent of the controls had good knowledge of ART medication and its perceived effectiveness compared to 58% of the cases. However, 7% of the controls had poor knowledge while 21% of cases had poor knowledge of ART medication and its perceived effectiveness.

### Independent Risk Factors of First Line ART Failure among People on Antiretroviral Therapy in Bulawayo City, 2016

Significant Independent risk factors associated with treatment failure among PLWHIV on Antiretroviral Therapy in Bulawayo City were: stopping medication when feeling worse (AOR= 3.5, 95% CI 1.8-7.0), missing medication the previous week (AOR=2.5, 95% CI 1.1-5.9), forgetting to carry medication when travelling (AOR=2.2, 95% CI 1.1-4.5) and having insufficient food on ART (AOR=3.2, 95% CI 1.8-5.7). Those who had disclosed to their partner were 45% less likely to have treatment failure.

## Discussion

The study established a strong association between non-adherence and first-line ART failure among People living with HIV in Bulawayo City. The finding was consistent with that of Matare et.al (2015) who found that those who had poor adherence were more likely to fail first-line ART^11^. This finding underscores the critical importance of strict adherence to medication regimens in effectively managing the condition and preventing disease progression.

Patients who stopped taking ART when they felt worse and never consulted the clinician were more likely to have treatment failure. Some of the study participants stated that they had a skin rash, nausea and vomiting which made them stop taking ART. These findings were similar to those of Chimbetete et.al (2011) who found that skin rash and other adverse effects were reported among the non-adhering clients^7^. Therefore, patients on ART should be educated on the possible side effects they would encounter during treatment and the importance of reporting these side effects to the clinician.

In this study, patients who forgot to take their ART medication when they travelled outside the country or to any area away from their original place of residence were at risk of treatment failure. A desk review of patient files showed that some patients who had defaulted treatment had travelled to countries such as South Africa or Botswana. The cessation of antiretroviral therapy (ART) among individuals who default after they have travelled to other countries poses a significant challenge to HIV management and treatment continuity. Several factors may contribute to the discontinuation of ART upon migration. These include disruptions in healthcare access and continuity of care, challenges in navigating healthcare systems in the destination country, cultural and language barriers, and logistical obstacles such as obtaining medication refills and accessing healthcare facilities. Additionally, stigma and discrimination related to HIV/AIDS may deter individuals from seeking care in their new environment.

This study identified insufficient nutritious food as a significant independent risk factor for treatment failure. The finding is biologically plausible as poor nutrition can weaken the immune system, compromise medication absorption and effectiveness, and exacerbate HIV-related symptoms and complications. Additionally, food insecurity may lead to inconsistent medication intake, as individuals prioritize meeting basic nutritional needs over adhering to their medication regimen. Chimbetete et.al (2011), also found that participants who had insufficient food while on first-line ART were more likely to have treatment failure compared to those who had a balanced diet^7^.

The study implicated that patients who drank alcohol and smoked marijuana were more likely to have first-line ART failure compared to those who did not. Substance abuse can disrupt medication adherence and exacerbate disease progression by compromising immune function and increasing the risk of opportunistic infections. People living with HIV who are on ART and drank alcohol or smoked marijuana were more likely to report missing medications because of forgetting^12^. Moreover, they are more likely to engage in risky sexual behaviour that can lead to reinfection or transmission of drug-resistant strains of the virus, further complicating treatment efforts^13^. This was stated by one of the key informants who stated that they engaged in unprotected sex with their client so that they could get money for school fees and rent.

The study established that patients who were initiated on ART having AIDS-related illness (WHO Stage 3 and 4) and had a low CD4 count were more likely to have treatment failure. Delayed initiation of treatment not only allows the virus to replicate and spread within the body but also increases the likelihood of developing drug resistance, limiting treatment options and efficacy in the long term. The study pointed out that some male patients had challenges in accepting their HIV status and would go for treatment when the disease had advanced. This was coherent with the findings by Kwobah et al (2012) who found that males who were initiated late were at an increased risk of treatment failure^6^.

In the study, the majority of cases had poor knowledge of ART compared to the controls. This was consistent with findings from a study done in Tanzania by Indindil et.al (2012) who found that the majority of controls had good knowledge of ART compared to the cases^14^. We also found that some clients were not sure of what to do when they missed their doses. This is also consistent with the findings by Almedia et.al (2009) who found out that the minority of respondents, 14%, knew that a missed dose was taken if it was not near the next dose^15^. Therefore if patients on ART do not understand the information given to them by the health care provider, they will not follow their medication as prescribed resulting in ART failure.

The study found that attending counselling before ART initiation was protective for treatment failure. This was consistent with the findings by Matare et.al (2015) who found that counselling was protective from treatment failure. However, it was noted that some patients stopped taking ART because of drug fatigue or would be taking other forms of traditional medicine besides ART; hence there is an increased risk of treatment failure^11^. Therefore, those who are HIV-positive need to attend counselling sessions before the commencement of ART as this will enable them to be aware of the possible side effects, and the importance of adhering to treatment.

## Study Limitations

This is a case-control study where the outcome has already occurred and therefore not possible to assess the temporal sequence of events. Therefore, it was difficult to ascertain if non-adherence alone could have led to treatment failure, as there are other causal factors such as acquired HIV drug resistance, health services factors or drug-related factors that can also lead to the development of treatment failure. Moreover, it was also difficult to ascertain if patient-related factors such as diarrhoea and nausea were a result of a lowered immunity due to treatment failure or were due to side effects. There was also differential recall bias from the study participants who could have forgotten events hence affecting the measure of association. To overcome that the researcher had to verify patient records and also use information from key informants. Pill counts were not used to assess medication adherence.

## Conclusion

The study demonstrated that there was a strong association between non-adherence and first-line ART failure among People Living with HIV on Antiretroviral Therapy in Bulawayo City. Additionally, stopping medication was strongly associated with treatment failure and cases had poor knowledge of ART as compared to the controls. Therefore, it is recommended that routine monitoring at every clinic visit of People Living with HIV on Antiretroviral Therapy should be done to monitor the client's adherence to medication and any adverse drug reactions that may affect adherence by looking at their CD4 count and viral loads. Assessment of pill counts should be done each time the clients come for their resupply to monitor their adherence. Health education on risky behaviours such as drinking alcohol, unprotected sex, smoking or taking hard drugs, should be reinforced when clients come for resupply. HIV counselling and testing including disclosure of results to couples should be promoted at the community level. There is also a need to provide alcohol reduction counselling to those who report current use as part of routine care. Moreover, health education sessions on HIV should be cascaded to schools, workplaces and other areas where people congregate. Further research shuld focus on assessing perceived family support and antiretroviral adherence among HIV-positive patients on ART in Bulawayo City.

## Public Health Action

The researcher presented key results to clinicians and representatives of PLWHIV and awareness campaigns on adherence to ART were done to PLWHIV.

## Figures and Tables

**Table 1:** Demographic Characteristics of study participants in Bulawayo City, 2016

Variable	Category	Cases (%)	Controls (%)	P value
Gender	Male	74(48.37)	79(51.63)	0.29
	Female	95(51.35)	90(48.65)	
Marital status	Single	36(61.0)	23(39)	0.1
	Married	89(44.7)	110(55.3)	
	Divorced	22(56.4)	17(43.5)	
	Widowed	22(53.7)	19(46.3)	
Education level	Secondary	139(50.73)	135(49.27)	0.29
	Tertiary	30(46.88)	34 (53.13)	
Place of residence	High density	157(52.3)	143(47.7) 26(68.4	0.01
	Low density	12(31.6		
Age in years	Median age	35(Q1=30, Q3=41)	39(Q1=33,Q3=47)	
Income level	Median	100(Q1=60,	100(Q1=75,	
	Income	Q3=200)	Q3=200)	

**Figure 1:**
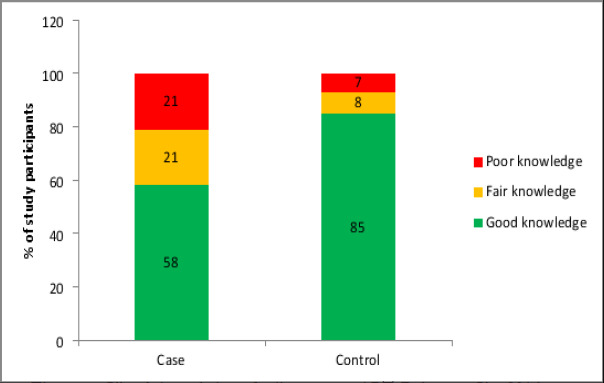
Client's knowledge of adherence to ART, Bulawayo City 2016

## Data Availability

The data that support the findings of this study are available from the Ministry of Health & Child Care Zimbabwe, but restrictions apply to the availability of these data. Data are however available from the authors upon reasonable request and with permission from the Ministry of Health Child Care Zimbabwe.

## List of Abbreviations

ART: Antiretroviral Therapy
IEC: Information, Education and Communication
